# Perceptual odor qualities predict successful odor identification in old age

**DOI:** 10.1093/chemse/bjac025

**Published:** 2022-11-05

**Authors:** Robert Lindroos, Rohan Raj, Stephen Pierzchajlo, Thomas Hörberg, Pawel Herman, Sandra Challma, Thomas Hummel, Maria Larsson, Erika J Laukka, Jonas K Olofsson

**Affiliations:** Gösta Ekman Laboratory, Department of Psychology, Stockholm University, Stockholm, Sweden; Gösta Ekman Laboratory, Department of Psychology, Stockholm University, Stockholm, Sweden; Gösta Ekman Laboratory, Department of Psychology, Stockholm University, Stockholm, Sweden; Gösta Ekman Laboratory, Department of Psychology, Stockholm University, Stockholm, Sweden; Division of Computational Science and Technology, KTH, Royal Institute of Technology and Digital Futures, Stockholm, Sweden; Gösta Ekman Laboratory, Department of Psychology, Stockholm University, Stockholm, Sweden; Smell and Taste Clinic, Department of Otorhinolaryngology, TU Dresden, Dresden, Germany; Gösta Ekman Laboratory, Department of Psychology, Stockholm University, Stockholm, Sweden; Aging Research Center, Department of Neurobiology, Care Sciences and Society, Karoliska institutet, Stockholm, Sweden; Gösta Ekman Laboratory, Department of Psychology, Stockholm University, Stockholm, Sweden

**Keywords:** aging, olfaction, perception, psychophysics

## Abstract

Odor identification is a common assessment of olfaction, and it is affected in a large number of diseases. Identification abilities decline with age, but little is known about whether there are perceptual odor features that can be used to predict identification. Here, we analyzed data from a large, population-based sample of 2,479 adults, aged 60 years or above, from the Swedish National study on Aging and Care in Kungsholmen. Participants performed both free and cued odor identification tests. In a separate experiment, we assessed perceived pleasantness, familiarity, intensity, and edibility of all odors in the first sample, and examined how odor identification performance is associated with these variables. The analysis showed that high-intensity odors are easier to identify than low-intensity odors overall, but also that they are more susceptible to the negative repercussions of old age. This result indicates that sensory decline is a major aspect of age-dependent odor identification impairment, and suggests a framework where identification likelihood is proportional to the perceived intensity of the odor. Additional analyses further showed that high-performing individuals can discriminate target odors from distractors along the pleasantness and edibility dimensions and that unpleasant and inedible odors show smaller age-related differences in identification. Altogether, these results may guide further development and optimization of brief and efficient odor identification tests as well as influence the design of odorous products targeted toward older consumers.

## Introduction

Olfactory abilities are important for health and well-being, as olfactory sensations facilitate appetite and food intake and prevent us from inhaling hazardous chemicals or ingesting spoiled food (Croy et al. 2014; Köster et al. 2014; Olofsson et al. 2021). Despite human olfaction being highly sensitive in most adults, a reduced sense of smell is nevertheless common in aging. In a recent meta-analysis, olfactory impairment was estimated to have a prevalence of 22% in the adult population (Desiato et al. 2021). Olfactory abilities decline in old age and olfactory impairment is more prevalent in older adults (Doty et al. 1984a; Larsson et al. 2016; Seubert et al. 2017). An accelerated age-related olfactory loss may be an early sign of cognitive and neurological disorders, including Alzheimer’s disease and Parkinson’s disease (Doty 2012; Rahayel et al. 2012; Kotecha et al. 2018), and it may signal an increased mortality risk (Pinto et al. 2014; Ekström et al. 2017; Pang et al. 2022). Both central and peripheral olfactory processes are affected by aging (Seubert et al. 2020; Fitzek et al. 2022) but the evidence is yet scarce regarding the specific factors underlying successful odor identification. A specific aim in the current study is therefore to elucidate the role played by perceptual features for the identifiability of common test odors.

The most common method for assessing the olfactory system is odor identification tests. A variety of identification tests are available (Doty et al. 1984b, 1996; Hummel et al. 1997; Nordin et al. 1998; Jackman and Doty 2005), and they have in common that participants are presented with common odors and are asked to select the correct source name from a list of alternatives. Typically, several odors are presented and a sum score of successful identifications is calculated. Odor identification tests are easy to administer and the outcome is often used as an index of olfactory ability. Identification is quicker to assess than olfactory detection thresholds, and also provides a valid assessment of ecological olfactory functions. The downside of using odor identification as the only assessment of the integrity of the sense of smell test is that poor performance has unknown origins. For example, performance impairments may be due to low olfactory sensitivity, impairments in quality discrimination, and/or associating and retrieving the corresponding verbal label. In aging, odor identification deficits might be a consequence of a combination of sensory (e.g. odor threshold, discrimination), and cognitive impairments (fluency, vocabulary; Larsson et al. 2004, 2005; Olofsson et al. 2009). In Alzheimer’s disease, odor identification scores are more impaired than those of sensory-level olfactory tests, suggesting a predominantly odor-cognitive impairment (Larsson et al. 1999; Rahayel et al. 2012; Murphy 2019).

The odors included in identification tests are often selected with the overall aim to be easily identified by healthy individuals with a normal sense of smell—a prerequisite for establishing those who have an olfactory impairment. This means that odors should reach a certain intensity level in order to reach a suprathreshold level. They should also be familiar to the community of patients and participants, provide a reasonable match with only one of the response alternatives, and span a broad range of olfactory qualities (Doty et al. 1984b; Hummel et al. 1997). The distractors are typically selected to be distinct from the targets (Nordin et al. 1998). The more distinct the alternatives are from the target odor, the higher performance levels are observed (Lötsch et al. 2021) Some tests are further designed to be valid in a specific culture (Nordin et al. 1998), while others have been validated across different cultural settings (Doty et al. 1996; Hummel et al. 1997).

In odor identification tests, little is known about item-level performance differences, and to what extent perceptual features make some odors easier to identify than others. The most important odor-perceptual features are hedonic value (pleasantness), familiarity, intensity, and edibility (Doty et al. 1984b; Khan et al. 2007; Zarzo 2008; Mainland et al. 2014). The extent to which the odor activates the trigeminal system is also an important feature (Lötsch et al. 2021), but for most odors, trigeminal activation is apparent only at higher odor magnitudes, and thus trigeminality is expected to overlap with intensity. A few attempts have been made to quantify how the identifiability of specific odors may vary along these dimensions, but knowledge is yet scarce. In the most ambitious study to date (Konstantinidis et al. 2006), odor identification was assessed for 16 odors in a sample of 472 adult participants, and performance levels were predicted from perceptual odor features: familiarity, intensity, and hedonics. The authors found that the hedonic value of an odor can explain the decrease in identification, such that unpleasant odors are more stable in aging (Konstantinidis et al. 2006). However, the participants in that study were relatively young, as only 25 of the participants were older than 65 years old (the total range was 18–79 with a mean age of 41.1 years). Since most age-related decline in odor identification capability occurs after the age of 65 (e.g. Doty et al. 1984a), the question of which perceptual odor characteristics are associated with identifiability and its relation to old age is unclear. Understanding the odor-perceptual basis of odor identification deficits is a fundamental issue, since most olfactory dysfunctions are strongly associated with old age and identification tests are commonly used (Desiato et al. 2021; Olofsson et al. 2021).

Here, we use a large dataset (Larsson et al. 2016) with 2,476 participants in the age range of 60–100 years to investigate how odor identification is related to perceptual odor features. In previous research, this dataset has been used to understand risk factors for olfactory dysfunction (Palmquist et al. 2020), and the relationship between olfactory and cognitive dysfunction (Olofsson et al. 2020), using aggregated odor identification scores. The aim of the present study was to understand odor identification performance using odor *item* information as a function of perceptual dimensions. A strength of the current study is that the analyses are based on 2 different retrieval formats in the assessment of identification; free identification (naming the odor without cues) and cued identification (provision of cues). It is well established that free odor identification is much more difficult than cued odor identification. Also, the 2 types of retrieval processes engage different cognitive processes and cortical networks, and may therefore exhibit distinct clinical potential (Olofsson et al. 2013; Olofsson and Gottfried 2015). The results from the 2 tasks could be compared, since the same odors were used in both tasks.

## Materials and methods

Two sources of data were used. Study 1 was based on data from the baseline assessment of a prospective aging study in the Swedish community (The Swedish National Study on Aging and Care in Kungsholmen, SNAC-K). Study 2 used data obtained in a laboratory experiment at Stockholm University where perceptual ratings were collected for the 16 odors used in the SNAC-K study (licorice, pineapple, petrol, banana, rose, apple, cinnamon, mushroom, fish, coffee, leather, cloves, peppermint, lemon, garlic, and turpentine).

### Study 1

The study characteristics have been described in detail before (Larsson et al. 2016). A short summary is provided below.

#### Participants

**Fig. 1.**
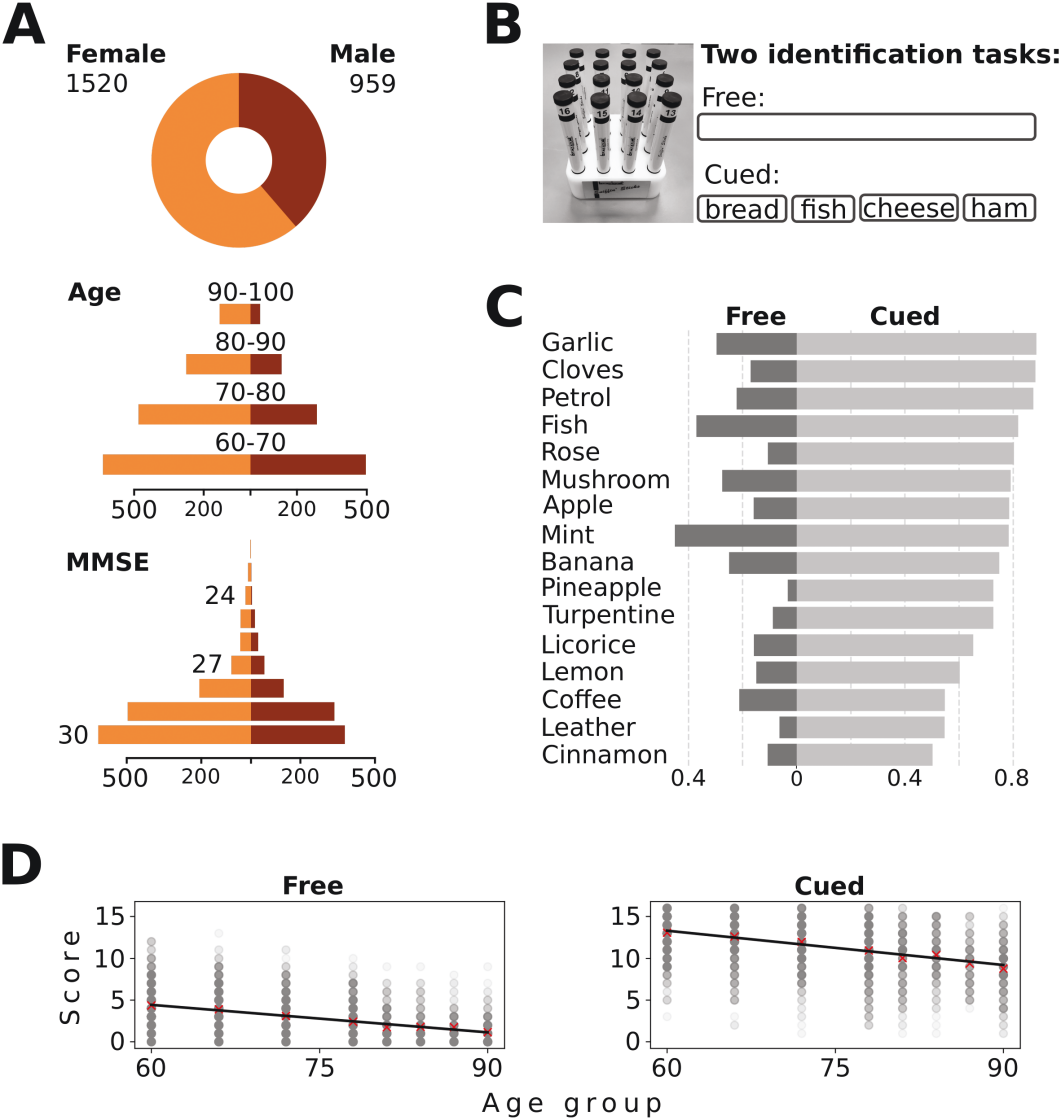
Overview of SNAC-K data. In A, the gender, age, and MMSE distributions of the participants are shown. In B, the sniffing sticks used in the testing are shown together with an illustration of the 2 odor identification tasks: free (no alternatives) and cued identification (1 target, 3 distractors). In C, the mean scores (proportion correct) for individual odors are shown for the 2 tasks. In D, the overall score across all 16 odors is shown for individual participants. The straight black line shows the regression trend over age used to distinguish high versus low performers in each age category.

Participants (*n* = 2,569) between 60 and 100 years of age underwent the olfactory assessment. See Fig. 1A for an illustration for the distribution of age and gender in the sample. Of these, 90 participants were excluded due to being diagnosed with dementia (*n* = 60), Parkinson’s disease (*n* = 20), schizophrenia (*n* = 9), or developmental disorder (*n* = 1). Out of the 2,479 remaining participants, 959 were male (39%) and 1,520 female (61%). Participants had, on average, a high Mini-Mental State Exam score (MMSE; *M* = 28.9, SD = 1.5). The distribution of MMSE as a function of gender is shown in Fig. 1A. Apart from dementia screening, we did not remove participants based on a cognitive cutoff value, because some level of cognitive deficit is expected in old age, and truncating the sample further might skew our results to deviate from the population values. Participants with an age equal to or over 90 years old were merged into 1 group due to a relatively small sample size.

#### Procedure

The odor identification test used in study 1 was the Sniffin’ TOM, a slightly modified version of the *Sniffin’ Sticks identification test* (Hummel et al. 1997). Compared with the original identification test, 2 of the 16 original odors were replaced, due to similarities with other odors in the set (i.e., orange, being similar to lemon, was replaced by petrol, and anis, being similar to licorice, was replaced by mushroom, see Croy et al. 2015; Larsson et al. 2016). Odor identification, in the form of free and cued identification, was examined in conjunction with an odor recognition memory test. The recognition memory data were not analyzed in this study. In the free identification task, participants were asked to name the odor without any cues. If the participant was unable to provide a name or if an answer was wrong, 4 written alternatives were presented and the participant was asked to select one of them. The 3 distractors associated with each target odor were predefined (for a table of distractors, see table 1 in Croy et al. (2015)). The olfactory testing itself was part of a neuropsychological assessment, which lasted about 2 h, and participants also underwent a physical and psychiatric evaluation, for a total testing time of about 6 h.

The final scores were coded as binary values on each trial, 1 for correct and 0 for incorrect. Assuming that a correct response in the free identification task would also give a correct response in the cued identification task, these 2 columns were here summed to derive the final cued identification score used in the analysis. In other words, if a participant was presented with fish odor and could identify it as “fish” without even viewing the 4 response alternatives (i.e. bread, fish, cheese, ham; see Fig. 1B), it was assumed that the participant would also choose “fish” among the response alternatives (Larsson et al. 2016). Responses that were perceptually close, but not identical, to the target in the free identification task were also considered correct—for example, crab, shrimp, and herring would also be considered as correct responses. The coding was conducted on a case-by-case basis by experts (Larsson et al. 2016). For an overview of the identification performance across each odor see Fig. 1C.

#### High versus low performers

Given that olfactory abilities are highly variable, individuals with high performance might resolve the odor identification tasks differently from those with a low level of performance. Such information might be of use in clinical contexts, for example when dissociating odor identification impairments in early stage dementia from those occurring in normal aging. To approach these issues, we applied an age-dependent split into high versus low performers. In order to adjust for noise in individual age groups, a fitted linear regression line was used as threshold rather than the mean in a specific group. For most groups the line and mean value was very close, but for the oldest participants there was a bias toward high performers. That is, most participants older than 95 years performed better than expected based on the linear regression although these groups were all merged into the 90+ group, as previously described. The scores of individual participants over the 2 tasks are shown in Fig. 1D.

#### Age effects in identification of single odors

In order to quantify the effect of age on single odors, a linear fit was made to the raw data (coded as binary values) as a function of age. This method facilitates noise correction in individual age groups, as single groups are restricted by all the data in the set. On the other hand, it does not account for nonlinear trends. The mean and SD of the variance explained by the linear fit were *r*^2^ = 87 ± 10% and *r*^2^ = 88 ± 8%, for *free* and *cued* identification, respectively.

#### Missing data

Some participants had missing data for 1 or more odors due to a failure to perceive the odor, refusal, or coding error (Larsson et al. 2016). Such occurrences were treated as a failure to identify the odor. Missing data accounted for only 1.3% of the total number of responses.

### Study 2

As noted, in order to further characterize the odors in the SNAC-K dataset, additional data regarding the perceptual features of each odor were collected in a separate psychophysical experiment. These data were additionally complemented by 2 previously published ratings for the relevant odors, in order to achieve greater reliability (Hummel et al. 1997; Shepherd et al. 2017).

#### Participants and procedure

**Fig. 2.**
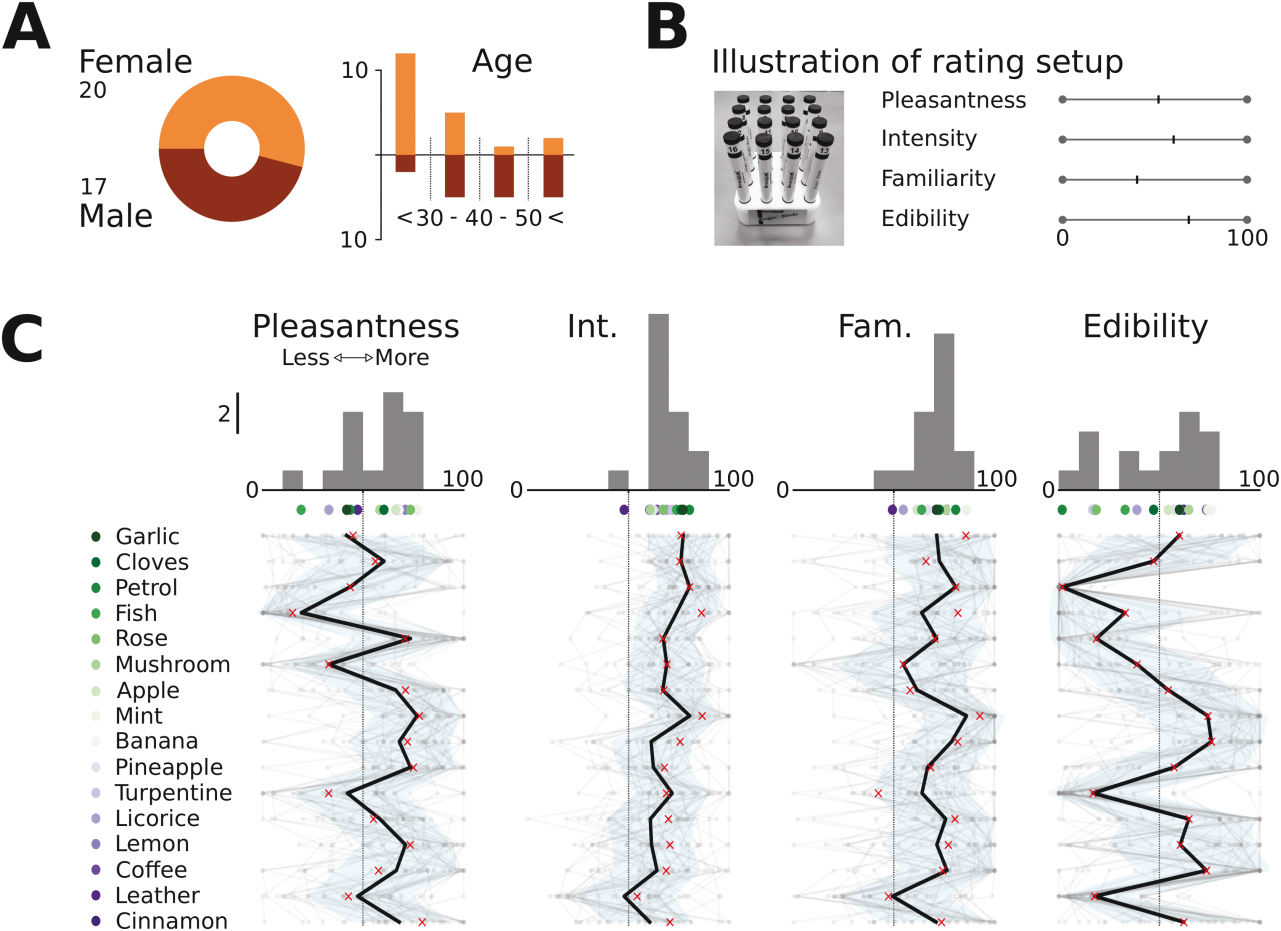
Overview of the perceptual ratings data: the distribution of gender and age of the participants who rated the perceptual features of the 16 odors (A), the rating setup (B), and the rated perceptual features (C). Light gray lines in the bottom row of panel C correspond to individual participants and the thick black line to the mean for each odor. Crosses show the weighted means calculated from the data shown here, and the previously published data (Hummel et al. 1997; Shepherd et al. 2017). The upper row in panel C shows the distribution of mean values for each perceptual feature—the scale is shared over the 4 panels. The dots under the panels show the underlying mean values. The shades of the dots correspond to the shaded dots left of the odor names. Odors are sorted according to proportion correct in the identification task in SNAC-K, see Fig. 1C. Int and Fam are abbreviations for intensity and familiarity, respectively

The participants consisted of 37 adults in the age range 19–61 years (20 females and 17 males, see Fig. 2A) with self-reported normal olfactory abilities. Since the goal was to provide precise and accurate estimations of the differences between odors, we did not engage older raters who might have an age-associated olfactory impairment. Here, the participants were asked to rate all the 16 odors included in the SNAC-K, one by one, on the perceptual features: *pleasantness*, *intensity*, *familiarity*, and *edibility*. The ratings were done on a visual analog scale of 0–100 (see Fig. 2B for an illustration). The order of the presentation of odors across each individual participant was randomized. This was done in order to minimize order-dependent interference between the ratings of different odors. Participants completed the experiment alone in a quiet and well-ventilated testing room and were guided by written instructions on a computer screen. All ratings were conducted in a self-paced manner. Participants were asked not to wear any scented products during the procedure in order to avoid odor contamination. The rating procedure was followed by a pairwise similarity rating assessment, but data from this latter assessment were not used here. All subjects provided written informed consent prior to testing and were compensated with a 200 SEK voucher. The protocol followed the guidelines of the Declaration of Helsinki.

#### Pooling data

In order to increase the statistical power and robustness of the perceptual ratings, 2 additional datasets were also included from previously published articles using the Sniffin’ Sticks identification test. A weighted mean value was then calculated based on all the data and used in the analysis. However, the locally collected data also comprised edibility ratings and perceptual ratings of petrol and mushroom odors. The first of the incorporated datasets was shared by the authors of an earlier publication (Shepherd et al. 2017), and in the second, data was extracted from the original publication of the Sniffin’ Sticks odor identification test (Hummel et al. 1997).

For each odor the weighted mean was calculated across studies with respect to the number of participants in each study (*n*_1_ = 37, *n*_2_ = 74, *n*_3_ = 63), using the formula: $\frac{1}{n_{1}+n_{2}+n_{3}}\cdot sum_{\left(i=1-3\right)}[n_{i}\cdot odor_{x}rating_{i}]$. Here, *x* denotes the index of the 14 odors that were part of all 3 sources. Since ratings of the odors *mushroom* and *petrol* and the perceptual feature *Edibility* were unique for study 2, corresponding mean values were calculated without weighting. In one of the datasets (Shepherd et al. 2017), the range of the rating scale was 1–5, rather than 0–100, as in the other 2 datasets. To reach coherence between datasets, ratings were converted to 0–100 using the formula: $100\times (mean_{odor\text{\textasciitilde{}}\text{-}x}-1)/(5-1)$, where the mean was calculated individually for single odors. Also, as familiarity was not reported as a continuous variable by Hummel et al. (1997), it was excluded from the weighted familiarity.

### Statistics

Correlation statistics was calculated as the Pearson’s correlation coefficient using the *Pearson* method of the python library *scipy*. Linear regression was calculated using the *linregress* method of the same library (Virtanen et al. 2020).

Since the dependent variable in the data was binary, logistic regression was used to combine features. The R package glmer (Bates et al. 2015) was used to set up models of free and cued identification, respectively. For these models, the weighted mean values of the perceptual features were *z*-scored (normalized to zero mean and unity variance) and used as independent variables, together with the age and gender of participants. Models were also created without the age and gender variables, with similar results.

### Data availability

All locally collected data are available together with scripts for analysis and visualization through the following link: https://osf.io/nesyq/.

## Results

### Predictors of free and cued identification

**Fig. 3.**
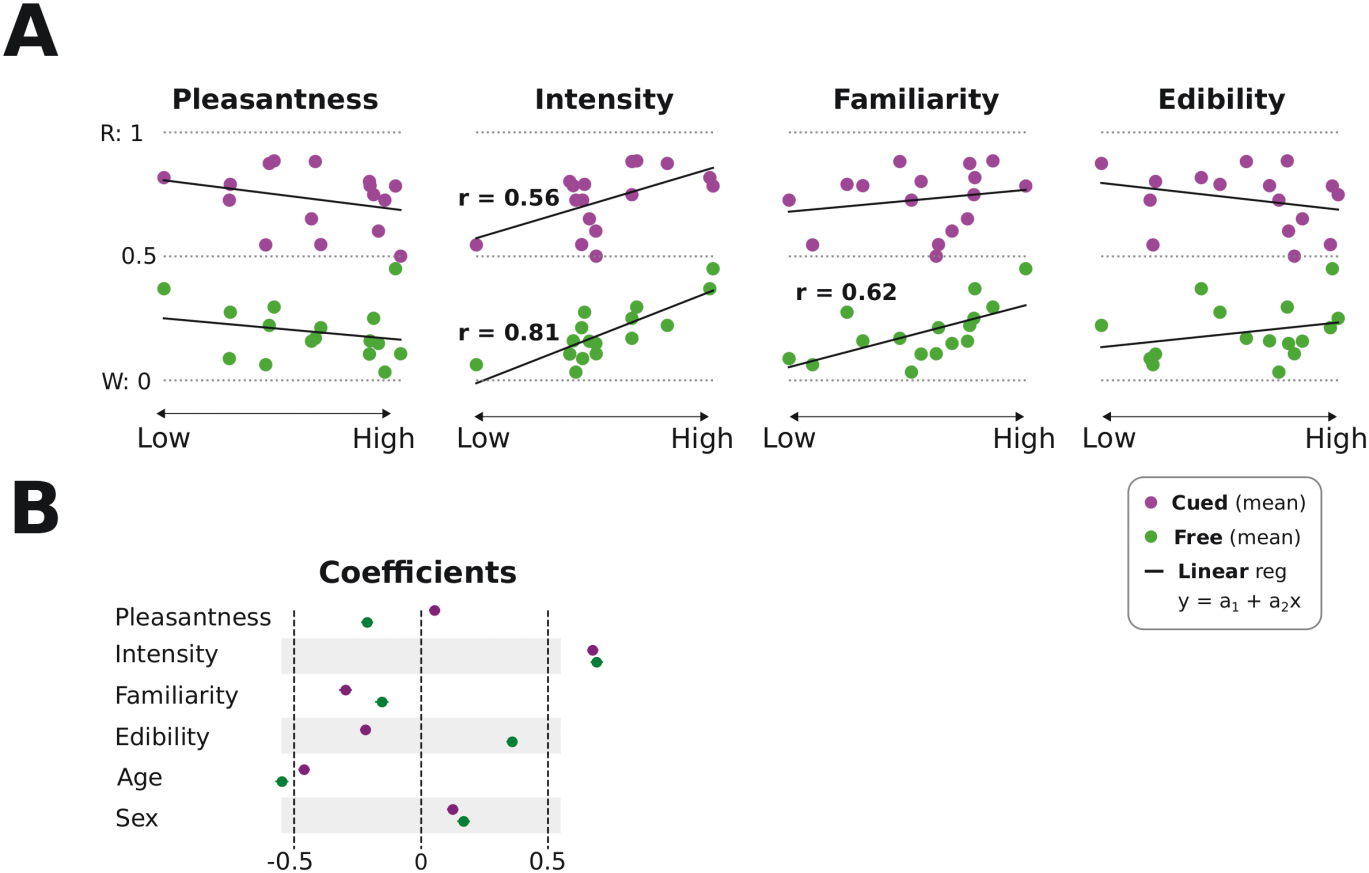
Predicting identification performance based on perceptual features. *A* shows the correlation between the perceptual features and performance. Significant correlations between identification and perceptual features are also stated directly in the respective panels. *B* shows the coefficients of a logistic regression model used to combine the perceptual features.

The odors varied on all perceptual attributes. In general, the ratings of intensity and familiarity were somewhat more restricted in range than pleasantness and edibility, as shown in Fig. 2C. This was expected, because weak and unfamiliar odors were not considered in the original Sniffin’ Sticks identification and the Sniffin’ TOM assessments (Hummel et al. 1997; Larsson et al. 2016). To address our key research question, we first correlated the mean identifiability of the odors with each of the 4 perceptual features: *pleasantness*, *intensity*, *familiarity*, and *edibility*. The results showed that *familiarity* and *intensity* were significantly correlated with *free* identification (*r* = 0.62, *P* = 0.01 and *r* = 0.81, *P* < 0.001, respectively). Additionally, *intensity* was correlated with *cued* identification (*r* = 0.56, *P* = 0.02, see Fig. 3A). In contrast, neither *edibility* nor *pleasantness* were reliably correlated with free or cued identification performance (*P* > 0.3). In the next step, the 4 perceptual dimensions were submitted as predictors with odor identification as criterion in a logistic regression model that also accounted for participants’ age and gender. Again, intensity was the strongest predictor of identifiability over the full dataset (Fig. 3B). Corroborating previous evidence (Larsson et al. 2004, 2016), the model showed that women were slightly more accurate in free and cued odor identification than men, and that identification performance decreases with age.

### Age-related effects

**Fig. 4.**
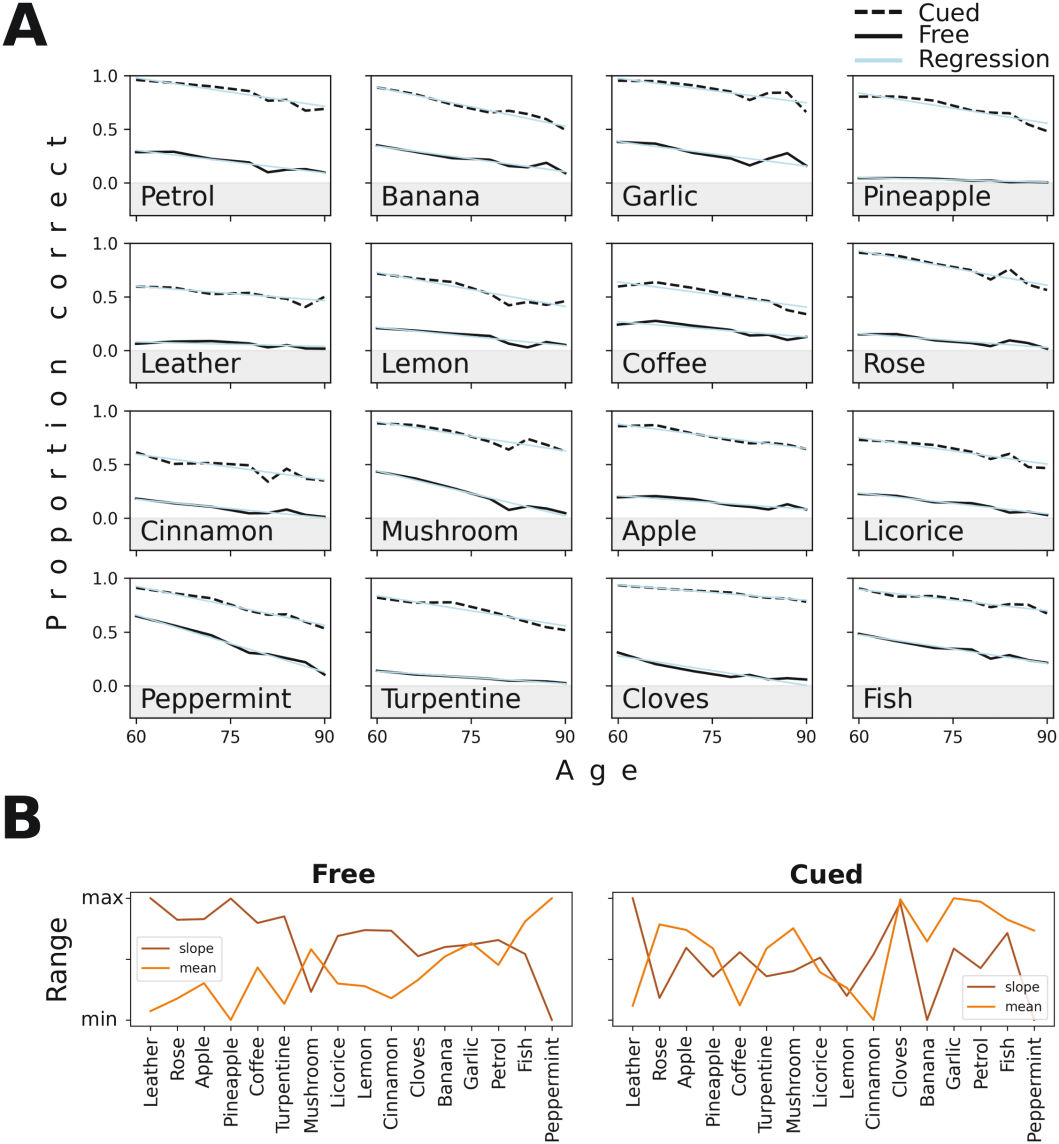
Performance over age in SNAC-K. *A* shows age differences in the identifiability of single odors with age. Solid and dashed lines show *free* and *cued identification*, respectively. Pale lines are linear fits. In *B*, the mean proportion and slope of the linear fits to the identifiability of single odor over age (cf. A) are shown. For comparison purposes, the values are here scaled to the range 0–1. The odors are also sorted by rated intensity with leather rated as the least intense and peppermint rated as the most intense.

Given that odor identification was positively associated with high-intensity odors, it is of interest to examine whether the slopes of the gradual age-related impairments in free and cued identification were associated with intensity. The overall identifiability of the odors decreased as a function of participant age, both in free and cued identification, as was previously shown in the SNAC-K dataset (Larsson et al. 2016). However, the results of our current item-level analysis showed that the identifiability of some odors decreased more than others (Fig. 4A).

When we quantified these trends by correlating perceptual features with the effect of age on single odors, we found a clear but somewhat surprising pattern: odors with higher intensity showed larger age decreases for *free* identification (*r* = −0.69, *P* = 0.003). The age-associated free identification decrease was also negatively correlated with the mean identifiability of each odor (*r* = −0.85, *P* < 0.001, Fig. 4B). That is, more intense odors are easier to identify overall, but they are also more susceptible to the negative repercussions of age for *free* identification. A clear example of an odor with this characteristic is peppermint, which is the odor that shows the highest free identification rate, but also shows a steep decrement across age cohorts. At the age of 60, identification is about 60% whereas it decreases to almost zero among the 90-year olds. In contrast, the cued identification task did not show the same pattern. Here, the observed age-associated deficits were neither reliably correlated with intensity (*r* = −0.31, *P* = 0.2) nor the mean score of each odor (*r* = −0.08, *P* = 0.8, Fig. 4B).

### Effects of pleasantness on odor identification

Earlier research showed that cued identification of unpleasant odors is less affected by aging (Konstantinidis et al. 2006). In agreement with this result, the present findings show a trend that age differences for unpleasant odors were generally smaller than the ones for pleasant odors. However, the trend failed to reach a conventional level of significance (*r* = −0.47, *P* = 0.069).

### Analysis of high and low performers

**Fig. 5.**
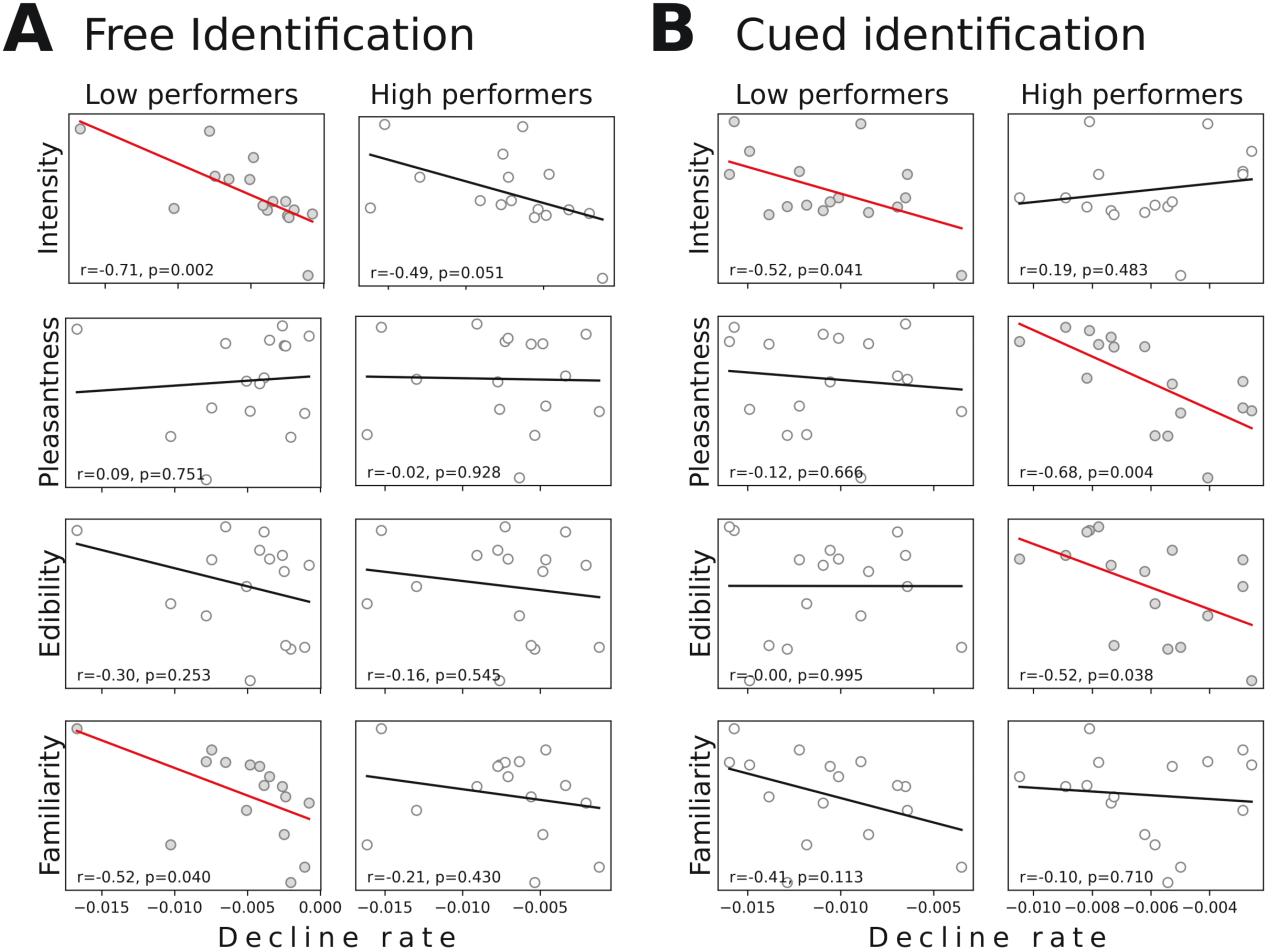
Identification of individual odors across age in high- versus low-performing individuals. The perceptual features are here plotted against the identification decline rate of single odors over age. A significant correlation is differentiated from a nonsignificant by gray and white markers, respectively (also the regression lines have different colors/shades in the two cases).

The total scores on *free* and *cued* identification show a large interindividual variation in all age cohorts (Fig. 1D). This raises the question whether high and low performers rely on different perceptual features in the odor identification tasks. In order to investigate this issue, we estimated the correlation of the age-associated impairment in performance with perceptual features separately in groups of high and low performers. For low-performing individuals, we once again observed that the identification of the more intense odors showed the largest age differences (*r* = −0.71, *P* = 0.002 and *r* = −0.52, *P* = 0.041 for free and cued identification, respectively). Also, more familiar odors had a larger age-associated decline for this group (*r* = −0.52, *P* = 0.040 and *r* = −0.41, *P* = 0.113), but for the high-performing individuals the correlation with intensity and familiarity was smaller (see Fig. 5). Interestingly, the cued identification performances of this group instead correlated significantly with pleasantness (*r* = −0.68, *P* = 0.004) and edibility (*r* = −0.52, *P* = 0.038). Thus, discrimination of unpleasant and inedible odors (e.g. turpentine) from distractors are relatively preserved in old age, compared with pleasant and edible odors (e.g. peppermint)—but this is overshadowed by intensity effects in low-performing individuals.

## Discussion

Despite olfactory loss being highly common, especially in old age, it is not well known which odors are functionally retained and which are not. Understanding which odor-perceptual features determine their perseverance in old age may help us better understand the aging olfactory system, and pave the way for olfactory assessments that may more efficiently discriminate between normal aging and age-related disorders.

Here, we identified the basic perceptual aspects associated with free and cued identification of odors in old age. The results indicate that the perceived intensity of the odor is an especially strong predictor of odor identification. If the perceived intensity is low, failure of identification will likely follow. This relation was particularly strong in the free identification task and hence independent of the relative similarity of target–distractors in the cued identification task. Although a certain level of odor intensity is obviously needed for identification, odor intensity variation has to our knowledge not previously been shown to constitute a major predictor of odor identification. Instead, prior research has indicated that odors with negative valences are preserved in odor identification among older adults, possibly due to an evolutionary advantage of avoiding hazardous exposure (Konstantinidis et al. 2006). Intensity differences were, however, not assessed for the odors assessed in those studies, and our results thus complement previous research.

Our analyses of the age-associated impairments, from the youngest cohort (i.e. 60 years old) to the oldest (i.e. over 90 years old) cohort, gave further insights into how odor identifiability depends on perceptual features. Here, the most intense odors (which were most easily identified in the younger cohorts) showed the most pronounced age-related differences in the free naming task. This indicates a model where odor identification over a population is proportional to the perceived intensity of the odor. We interpret this finding as likely reflecting a diminished olfactory-sensory ability in the oldest adults. With diminished olfactory sensitivity, the odors that were once clearly perceived (in this case, the most intense) become more subtle. It is well established that old age is associated with decreased odor detection sensitivity (Stevens and Cain 1987), as well as decreased magnitudes and emotional intensities of perceived odors (Wysocki and Gilbert 1989; Vieillard et al. 2021). Sensory deficits are therefore, in our opinion, the most parsimonious explanation as to why the free identification advantage for high-intensity odors decreases in old age. Cued odor identification did not yield the same pattern, perhaps because the written cues enable participants to use top-down processes, such that they recover an odor representation that may only be partially activated by the odor in isolation. On a cortical level, odor identification cues can activate olfactory and associative cortical networks to facilitate odor identification performance (Olofsson et al. 2012, 2014; Zhou et al. 2019).

Dividing the sample into high and low performers yielded further results that indicated how these 2 subgroups may engage differently with perceptual odor features as they complete the identification tasks. Low performers appear especially affected by the impaired identifiability of high-intensity odors in older ages, similar to the sample as a whole. High performers, on the other hand, may process the odor stimuli differently. For them, results are similar to those observed in previous research, that unpleasant odors are less affected by aging (Konstantinidis et al. 2006). There is increasing evidence that unpleasant odors have a privileged access to the olfactory cortex (Iravani et al. 2021) and that there is cross-cultural agreement on which odors are unpleasant (Arshamian et al. 2022; Oleszkiewicz et al. 2022). We speculate that for older participants that have a sufficient olfactory capacity (high-performers), unpleasant odor representations are preserved.

Previous work has emphasized the distinction between intensity and identification; atrophy of the medial temporal lobe impairs identification but leaves intensity judgments intact (Eichenbaum et al. 1983; Jones-Gotman and Zatorre 1988). In healthy individuals, odor identification processes are primarily dependent on the hippocampus and orbitofrontal cortex (Kjelvik et al. 2012; Olofsson et al. 2014). These areas are located downstream from the primary olfactory cortices that process odor intensity (for review see Mainland et al. 2014). Our findings are, however, consistent with this distinction, as disruptions in the earliest olfactory processing stages are likely to have negative consequences also for identification. Our results show that among the perceptual features assessed here, intensity differences among odors are the major perceptual predictor of odor identifiability. This finding adds to previous evidence that cued identification performance is dependent on the response alternatives, and the interactions between the odor percept and memory representations they evoke (Gudziol and Hummel 2009; Negoias et al. 2010).

Free identification is also dependent on an additional odor feature, namely high familiarity. Free odor identification does not involve any verbal/visual cues or response options, making odor name retrieval more challenging. Highly familiar odors are needed for this task, likely because familiar odors have a robust memory representation, which makes them more retrievable (Stevenson and Mahmut 2013). Interestingly, the availability of response options in the cued identification task appears to reduce the need to use very highly familiar odors, presumably because the participants use the provided odor labels as effective memory retrieval cues.

In characterizing the perceptual variability of the odor set, we decided to use adult raters without self-reported olfactory dysfunction. This group of raters was not age matched with the older participants who participated in the odor identification test. We reasoned that since the goal of the rating experiment was to accurately capture differences between the stimuli, it was not preferred to use older raters, since they would have, on average, a poorer olfactory capacity.

In sum, our results imply that odor identifiability varies across test odors, and that this variability partly can be explained by perceptual odor features. Most important of these features is intensity, which confers a high overall identifiability (both in free and cued identification tests), although this advantage declines rapidly in old age. High-performing individuals may, however, retain an ability to identify unpleasant odors from inedible sources. These factors can be exploited in the design of specific purpose olfactory tests. Specifically, odor identification tests with a reduced number of odors have been proposed as rapid screening tests for Alzheimer’s disease dementia and Parkinsons’ disease (Tabert et al. 2005; Woodward et al. 2018; Joseph et al. 2019; Auger et al. 2020). However, there has been no consideration of the perceptual factors that would guide odor selection for such purposes. Future research may address whether these different clinical groups might be impaired on different perceptual odor features. If this hypothesis is confirmed in future research, odor intensity, pleasantness, and edibility could be considered when selecting odors for rapid identification tests aimed at differentiating between, for example, healthy older adults and those with an increased risk of developing dementia (Payne et al. 2022). Understanding which odors are most identifiable in older adults can also be useful in developing consumer products for older consumers.

This study has limitations. First, despite benefitting from a very large sample on odor identification in older adults, we had no data on detection or discrimination ability, which would have allowed us to explore sensory influences on odor identification. Older people generally score lower on tests of detection threshold which further highlights sensory decline as a prominent source of olfactory dysfunction in old age (Hummel et al. 2002; Oleszkiewicz et al. 2019). Furthermore, we have not distinguished trigeminal sensations from pure olfactory sensations in our stimuli. As in many everyday olfactory experiences, olfactory and trigeminal sensations seamlessly blend together, especially at high concentrations. Sensitivity of the trigeminal nerve also decreases with age (Murphy 1983), which might explain the large impairment observed in free identification of odors with a high trigeminal response, for example peppermint (see Fig. 4A). One further possibility is that intensity ratings are influenced by activation of the trigeminal nerve since peppermint is also rated as the most intense odor in the set (Fig. 2C). These limitations might be addressed in future work. Our methodology and results facilitate further item-level approaches to odor identification. The insights yielded about perceptual features that are systematically associated with odor identification performance can be used for development and optimization of odor identification tests. Specifically, reducing the number of test odors, and making testing sessions brief and effective in achieving particular clinical and research goals, is of interest. In such efforts, target intensity and target–distractor similarity along the pleasantness and/or edibility dimensions should be specifically considered in the design.
